# Reinterpretation, reclassification, and its downstream effects: challenges for clinical laboratory geneticists

**DOI:** 10.1186/s12920-019-0612-6

**Published:** 2019-11-29

**Authors:** Julia El Mecky, Lennart Johansson, Mirjam Plantinga, Angela Fenwick, Anneke Lucassen, Trijnie Dijkhuizen, Annemieke van der Hout, Kate Lyle, Irene van Langen

**Affiliations:** 1 Department of Clinical Genetics, University Medical Centre Groningen, Groningen, The Netherlands; 2 Clinical Ethics and Law Southampton, University of Southampton, Southampton, UK

**Keywords:** Reinterpretation, Reclassification, Genetics, Laboratory, Focus group

## Abstract

**Background:**

In recent years, the amount of genomic data produced in clinical genetics services has increased significantly due to the advent of next-generation sequencing. This influx of genomic information leads to continuous changes in knowledge on how genetic variants relate to hereditary disease. These changes can have important consequences for patients who have had genetic testing in the past, as new information may affect their clinical management. When and how patients should be recontacted after new genetic information becomes available has been investigated extensively. However, the issue of how to handle the changing nature of genetic information remains underexplored in a laboratory setting, despite it being the first stage at which changes in genetic data are identified and managed.

**Methods:**

The authors organized a 7-day online focus group discussion. Fifteen clinical laboratory geneticists took part. All (nine) Dutch clinical molecular genetics diagnostic laboratories were represented.

**Results:**

Laboratories in our study reinterpret genetic variants reactively, e.g. at the request of a clinician or following identification of a previously classified variant in a new patient. Participants currently deemed active, periodic reinterpretation to be unfeasible and opinions differed on whether it is desirable, particularly regarding patient autonomy and the main responsibilities of the laboratory. The efficacy of reinterpretation was questioned in the presence of other strategies, such as reanalysis and resequencing of DNA. Despite absence of formal policy regarding when to issue a new report for clinicians due to reclassified genetic data, participants indicated similar practice across all laboratories. However, practice differed significantly between laboratory geneticists regarding the reporting of VUS reclassifications.

**Conclusion:**

Based on the results, the authors formulated five challenges needing to be addressed in future laboratory guidelines: 1. Should active reinterpretation of variants be conducted by the laboratory as a routine practice? 2. How does reinterpretation initiated by the laboratory relate to patient expectations and consent? 3. When should reinterpreted data be considered clinically significant and communicated from laboratory to clinician? 4. Should reinterpretation, reanalysis or a new test be conducted? 5. How are reclassifications perceived and how might this affect laboratory practice?

## Introduction

In recent years, the amount of genomic data produced in clinical genetics services has increased significantly due to the advent of next-generation sequencing. This influx of genomic information not only leads to an increased diagnostic yield, but also results in a continuous redrawing of connections between genetic variants and genetic conditions. This is demonstrated by the fact that many variants have been reinterpreted and reclassified (Table 1) to either more or less pathogenic than previously thought [28] and new genes and variants are continuously identified to potentially play a role in the development of medical conditions [2, 16, 19, 26]. The changing nature of genetic knowledge can have important consequences for patients who have had genetic testing in the past, as new information on genetic variants may affect their clinical management. It has been investigated extensively when and how patients need to be recontacted when new genetic information becomes available, both in clinical and in research contexts ([5, 14, 15, 21]; Carrieri et al. 2019 [29]; David et al. 2019 [7, 12, 24];). However, the issue of how to handle the changing nature of genetic information in a laboratory setting is significantly underexplored [33], despite it being the first stage at which changes in genetic information are identified and managed. Research and guidelines that include laboratory geneticists’ perspectives on this topic focus on what to do *after* new genetic information has been generated. This focus omits critical questions regarding the process *before* and *during* which new information is identified: If, when, and how often should available evidence on the pathogenicity of genetic variants be reinterpreted by the laboratory after initial test results have been given to patients?; Who is responsible for making the decision to conduct reinterpretation?; And in which cases has knowledge around genetic variants changed sufficiently for a laboratory to reclassify them and issue a new report, informing clinicians of this change? Therefore, whilst acknowledging that recontact is an important topic within the laboratory context [29], this paper primarily focuses on laboratory practice around reinterpretation and reclassification.

**Table 1:** Key definitions

Reinterpretation	
Re-evaluation of genetic variants that have been analyzed and interpreted in the past, in order to assess whether the initial classification is still correct or should be changed in light of new information. This may result in an updated and modified report on the data in question by the laboratory to the clinician.	
Reclassification	
Assigning a different pathogenicity to a variant that has been classified in the past (e.g. from benign to likely pathogenic). In this article, we refer to the classification system described by Richards et al. [27] in which class 1 is benign; class 2 is likely benign; class 3 is variant of uncertain significance; class 4 is likely pathogenic; class 5 is pathogenic. Adapted from Carrieri et al. 2018	

To our knowledge, this is the first qualitative inquiry into perspectives of clinical laboratory geneticists regarding their current and desired future practice in the context of reinterpretation and reclassification of genetic variants. Based upon a week-long online focus group discussion with clinical laboratory geneticists representing all (nine) Dutch clinical molecular genetics laboratories, the authors identify key issues and concerns that laboratory geneticists may contend with in practice, in order to scope out challenging themes within an uncharted territory that should be further addressed in guidelines, research, ethical considerations, and practice.

## Methods

We invited the heads of department of all (*n* = 9) clinical molecular genetics diagnostic laboratories in the Netherlands to participate in a 7-day online focus group discussion in September 2017, hosted on the platform focusgroupit.com. We requested the heads of department to extend our invitation to all clinical laboratory geneticists in their laboratory. All laboratories were non-commercial, as, by law, only certified, non-commercial molecular genetics laboratories are allowed to conduct clinical genetic testing in The Netherlands. All laboratories were affiliated with clinical genetics departments in academic hospitals, which are responsible for all clinical genetics diagnostics conducted in The Netherlands and whose services in genetic testing and counseling are covered by public healthcare insurance. Fifteen clinical laboratory geneticists decided to participate in the discussion. All molecular genetics diagnostic laboratories in the Netherlands were represented in the sample (1–3 participants per laboratory). Each participant was allocated an alias with the aim to remain anonymous within the group.

A semi-structured approach to the facilitation of the discussion was chosen, so that new themes could emerge through discussion by and between participants. The discussion questions posed by the authors served to guide the discussion, simultaneously leaving room for participants to bring in their own themes and issues. A topic list consisting of questions and propositions (see Additional file 1) was composed to identify current and desired practice around reinterpretation and reclassification of variants, as well as laboratory communication of reinterpreted and reclassified data to clinicians. The topic list was designed by the research group, in which laboratory geneticists were represented, based on existing literature and clinical experience. At the beginning of each of the first 5 days of the online discussion, a new topic was posted on the platform. The topic list was adjusted iteratively as the discussion progressed, i.e. questions and propositions were adapted based on themes that came up in discussion on previous days.

Participants were invited to log in one or multiple times per day at their own convenience (asynchronous format), in order to respond to the questions posed and to each other’s responses. In the final 2 days of the discussion, no new topics were posted and participants were encouraged to respond, if they had not yet done so, to previous topics or to continue on-going discussion. The discussion was moderated by JM, a medical anthropologist, who asked follow up questions to each response. One laboratory geneticist, TD, and one clinical geneticist, IL, who were not participants, followed the discussion and advised JM on moderation and follow-up questions. After 1 week, the forum was closed and each participant received a voucher worth €25.

Thematic analysis [6] was conducted inductively with the help of NVIVO software by QSR International (version 11), focusing on challenges regarding reinterpretation and reclassification. JM and LJ, a bioinformatician with a background in ethics, coded the data independently. Subsequently all coding was compared and discrepancies were discussed until agreement was reached. Citations included in this paper were translated from Dutch to English by JM and LJ.

In an effort to be of most use to broader academic, policy, guideline, and practice discussions regarding reinterpretation and reclassification, we combined the results and discussion sections. This allowed for direct reflection on the findings in the context of current literature available on reinterpretation and reclassification.

## Results and discussion

During the focus group discussion, participants described their current and desired practice, whilst identifying issues regarding reinterpretation and reclassification. Further challenges came to light through discrepant practices described by participants. Based on these accounts, the authors identified the following five challenges.

### Should active reinterpretation of variants be conducted by the laboratory as a routine practice?

Participants indicated that in current Dutch clinical genetics laboratory practice, reinterpretation mainly takes place reactively, i.e. in response to an event (Table 2). There is no routine practice in Dutch laboratories of actively reviewing the latest available evidence on variants. In terms of possible, future implementation of active, periodic reinterpretation, a distinction can be made between what is desirable and feasible laboratory practice.

**Table 2:** Types of reinterpretation illustrated by fictional cases^a^

Reactive reinterpretation	
The laboratory conducts reinterpretation of previously identified variants due to an external trigger. This can be a) a request from patient or clinician or b) identifying a previously classified variant in a new patient	
Case a. Reactive reinterpretation upon request: *Ms. P’s 10 year old child, A, has learning difficulties. A is referred to Dr. F, a clinical geneticist, and a genetic test is performed, identifying a variant of uncertain significance (VUS/class 3) in FOXP2. Dr. F discusses this result with Ms. P, explaining that a genetic variant has been identified that could possibly be the cause for A’s learning difficulties, but could equally likely play no part. Dr. F recommends that Ms. P return to the clinic in a few years to enquire whether new information is available. Ms. P contacts Dr. F two years after the initial results, who in turn contacts the laboratory to inform about the current state of affairs regarding the FOXP2 VUS. The laboratory reinterprets the variant and informs Dr. F that the variant can now be reclassified as class 1 benign and therefore non-causal for A’s symptoms.*	
Case b. Reactive reinterpretation upon identification of variant in new patient: *Mr. C is diagnosed with colorectal cancer and is referred for genetic testing. A likely pathogenic variant (LP/class 4) in MSH6 is identified. Three years later a different patient, Ms. L, unrelated to Mr. C, is referred to the clinic due to colorectal cancer. A genetic test is performed and the same MSH6 variant is identified. Review of the evidence now suggests it is a pathogenic (class 5) variant and the classification in Mr. C’s case is also changed to pathogenic. A new report informing of this change is issued to Mr C’s clinician.*	
Active reinterpretation	
The laboratory initiates reinterpretation of previously identified variants, without an external trigger	
Case c. Active reinterpretation: * A molecular genetics laboratory decides to biannually reinterpret all variants that are classified as VUS in their database. When new evidence is sufficient, these variants are reclassified and their new classification is registered in the laboratory’s database. Clinicians whose patients have been identified to carry these variants are informed of the reclassifications. All patients whose genetic testing is conducted through this laboratory are informed of this policy at their clinical consultations.*	

^a^The fictional cases listed in this table were not used during the focus group discussion, but rather serve to clarify different possible reinterpretation scenarios for the purpose of this paper

#### Desirability

Active, periodic reinterpretation of variants could be an important strategy for ensuring patients are alerted to changing evidence surrounding genetic variants. Participants indicated that rare variants in particular are currently less likely to be reinterpreted, as the chance that they are detected in other patients is relatively small. One laboratory was investigating ways to implement active, periodic reinterpretation of all VUS in their in-house database. However, active, periodic reinterpretation was not unequivocally considered a desirable practice by participants; they expressed concerns whether such reinterpretation could be adequately understood and consented to by patients during the process of genetic counseling (see Methods). Active reinterpretation of patient data was not seen by participants as a responsibility of the laboratory, as the laboratory’s aim, similar to other medical fields, was seen as providing service at request, rather than actively initiating services. Therefore the main responsibility to initiate reinterpretation of genetic data was considered to lie with patients (in tandem with their clinicians).

*“We are service-oriented, in other words: at the request of the patient.” (Participant 11) “In my opinion it’s comparable to a doctor: they don’t re-evaluate their pictures and files every year either to see if there is any new information. (Participant 3).*

#### Feasibility

In terms of feasibility, the main factor impeding active, periodic reinterpretation according to participants was work load. Such a procedure was currently deemed to be financially and logistically unachievable, as the process of reinterpretation was seen as laborious and unamenable to complete automation. Participants noted that since different conditions require different kinds of evidence to classify variants as either benign or pathogenic, combined with the fact that laboratory geneticists differed in what they considered as adequate evidence for (re) classification, automating reinterpretation and reclassification was considered challenging.

*“As mentioned by many [others], automation is hard, because the correct application of data present in databases and literature is almost impossible to interpret in silico. For this, you’ll always need humans.” (Participant 2).*

*“Active [reinterpretation] of data is currently not feasible in terms of work [load]. ( …*) *Possibly, if [these processes] are more automated in the future, there will be more possibilities to do this (and to want [to do this]).” (Participant 3)*

Several guidelines indicate that laboratories do not have any responsibility to routinely reinterpret genetic data, so as not to be overloaded or compromised in other duties related to patient care [4, 18]. However, we propose that active, periodic reinterpretation does not necessarily need to be conceptualized in an all-or-nothing way: a middle ground may also be feasible. Actively reclassifying only those variants for which clear, new evidence is present regarding a pathogenic (or benign) effect could benefit some patients, without leading to extensive database and literature searches. In terms of bioinformatics, this would mean that the threshold to flag a variant for possible reclassification is set high. Commercial companies exist that provide active, periodic reclassification [22]. Tools such as InterVar [17], which use a formalized protocol, could possibly aid automation of the classification process. Furthermore, an infrastructure in which classifications and variant frequencies are automatically shared between laboratories [24], as well as an infrastructure to alert clinicians or laboratory geneticists to updated classifications, as discussed by Aronson et al. [1], could aid management of reclassified variants. The field of artificial intelligence could offer interesting opportunities in this context. In terms of comparing genetics services to other medical services which tend to offer services at request, rather than actively initiating them, it is important to note that genetic data differs from most other kinds of medical data, as it is constant throughout a patient’s life; only its analysis and interpretation changes. As the laboratory knows more about current variant classifications than clinicians, let alone patients [11], it is important to explore to what degree laboratories could and should have systems in place for systematic reinterpretation. Many variants, especially relating to patients with rare diseases and patients from ancestries underrepresented in the genomic evidence base [5], are found infrequently on a global level. Importantly, not conducting active, periodic reinterpretation might result in an inequitable service where some patients more than others are left unaware of new evidence, thereby posing an injustice issue.

### How does reinterpretation initiated by the laboratory relate to patient expectations and consent?

Participants indicated that, in current Dutch laboratory practice, variant reinterpretation is initiated by laboratory geneticists during the analysis of new patients, without updated consent from previous patients with the same variants (Table 2, case b). Concerns existed among laboratory geneticists regarding the autonomy of these patients, as well as the durability of consent. Even if the possibility of reinterpretation was discussed during initial counseling, participants felt unclear about patients’ ability to fathom that consent regarding reinterpretation of their genetic information is given for an undetermined period of time. This may result in unexpected and possibly, in that moment, unwanted recontact.

“*When the same VUS is detected in a new patient, it will routinely be evaluated again. The question whether patients desire [reinterpretation] does not play a role in my decision making. [ …] [Because] the patient is informed about this possibility and also gave consent. However, I do wonder if they realize that they can be contacted two years later.” (Participant 9)*.

Concerns particularly existed regarding patient expectations, understanding, and wishes in discussions regarding active reinterpretation.

*“Very rarely you have to send a corrected letter to the first patient, who thinks his/her [DNA] examination has been concluded and often is not looking for [new information]. Therefore, you should not be doing this routinely for each detected variant.” (Participant 6).*

*“It seems to me that it’s of main importance that the patient wants [active, routine reinterpretation]!” (Participant 9).*

We pose that for laboratory geneticists, an important conceptual difference between active and reactive reinterpretation might be the generation of new information (active reinterpretation) versus reporting of information that has already been generated (reactive reinterpretation and reclassification following identification of a previously detected variant in a new patient). They may feel a stronger moral responsibility to act on information that has already been generated, as in the case of reactive reinterpretation following identification of a previously detected variant in a new patient, in comparison to the moral responsibility to take the initiative in generating new information. This may explain why more hesitance was expressed regarding respecting patient autonomy in the context of active reinterpretation in comparison with reactive reinterpretation. However, from the perspective of the patient, this conceptual difference may not exist. For the patient, being recontacted based on reactive reinterpretation initiated by the laboratory does not differ from being recontacted based on active reinterpretation initiated by the laboratory. Therefore, implementing active, periodic reinterpretation does not diverge from current practice, in terms of the possible negative consequence of unexpected recontact from a patient perspective, except that it would likely occur on a larger scale.

It is important not to alleviate the moral distress around patient autonomy as expressed by laboratory geneticists by automatically precluding active, periodic reinterpretation. Rather, these concerns should be addressed through empirical inquiry into what general patient expectations and understanding are regarding reinterpretation of their genetic data and through investigation of proper means of counseling and consent suitable for the genomic era. This could include dynamic consent, allowing patients to halt or continue active reinterpretation of their data at their preference; a larger emphasis during pre- and post-test counseling on the possibility of change in genetic knowledge and its implications for (family) medical management; and the exploration of technological infrastructures that allow the possibility, if desired by patients, of not reinterpreting or reclassifying their data. Particularly, an important question is whether it is good practice to give patients the opportunity to broadly opt out of potentially medically actionable information, as this is a complex decision regarding information that cannot be envisaged accurately by patients at the time. A potential additional problem with this approach is that different family members carrying the same variant(s) might then give different kinds of consent, leading to new reports being issued for some family members in case of variant reclassification, but not for others. This may give rise to difficult and tense family situations, as well as different medical treatment and screening protocols within one family, begging the question whether patient choice in this context is feasible and desirable in practice.

### When should reinterpreted data be considered clinically significant and communicated from laboratory to clinician?

Participants pointed out that when patients and their clinicians ask a laboratory for reinterpretation of a previously reported variant, the laboratory communicates any new information or lack thereof to the clinician of the patient in question.

In contrast, participants indicated that when a variant is reclassified without prior inquiry by a patient or their clinician (e.g. when the variant is found in a new patient and reclassified for all individuals carrying this variant), communication of this new information from laboratory to clinician does not occur by default. Whether a reclassification potentially affects a patient’s (or their family’s) clinical management, i.e. its clinical significance, is the underlying principle of Dutch laboratories’ practices and decisions related to recontacting clinicians. Our focus group discussion suggests that laboratory geneticists might often feel the clinician is responsible for establishing which reclassified variants are clinically significant enough to warrant recontact.

*“Communicating [reclassified variants] to the clinician seems to me [ …*] *to precede over the clarity of medical relevance. The latter is namely the eventual responsibility of the medical specialist.” (Participant 9).*

In practice, however, laboratory geneticists also make decisions on this matter by deciding which reclassifications are of clinical significance and therefore need to be communicated to clinicians. Despite absence of formal policy, participants indicated that it is standard practice to communicate the following reclassifications to clinicians, in case reinterpretation took place without request by patient or clinician: any variant that is upgraded to (likely) pathogenic (e.g. likely benign to likely pathogenic), as well as (likely) pathogenic variants that are downgraded (e.g. pathogenic to VUS) (Table 3). An exception constitutes pathogenic variants that are downgraded to likely pathogenic. Often, these reclassifications are not reported to clinicians, as they are not expected to affect clinical management. However, practice differs most significantly for VUS that are downgraded to (likely) benign or vice versa: some laboratory geneticists do, whilst others do not communicate these reclassifications to clinicians, as laboratory geneticists differ in whether they consider these reclassifications as clinically significant.

**Table 3:** Current practice in communication of reclassified variants from laboratory to clinician

		Reclassificatsion				
		B (1)	LB (2)	VUS (3)	LP (4)	P (5)
Initial classification	B (1)		NO	Varies	YES	YES
	LB (2)	NO		Varies	YES	YES
	VUS (3)	Varies	Varies		YES	YES
	LP (4)	YES	YES	YES		YES
	P (5)	YES	YES	YES	Varies	

Variants are shown with their initial classification and after reclassification, ranging from class 1 to class 5 (representing benign, likely benign, variant of uncertain significance, likely pathogenic, and pathogenic)

Yes - This type of reclassification is communicated from laboratory to clinician

No - This type of reclassification is not communicated from laboratory to clinician

Varies - Communication of this type of reclassification from laboratory to clinician varies between laboratory geneticists

Some laboratory geneticists argued that when a VUS is reclassified to a benign or likely benign variant, this does not merit recontact with the referring clinician, as it would not affect patients’ medical management. This is in line with ACMG recommendations that VUS should not be used in clinical decision-making [27]. However, one participant observed that clinicians often communicate VUS to patients as *possibly* causative of their clinical symptoms, when they are detected and reported in the original laboratory report.

*“In case of a reclassification from (…*) *[class] 3 to 2/1, in principle no [new lab report] will follow, because it doesn’t change [clinical] management.” (Participant 8).*

*“I do report a reclassification from [class] 3 to 1/2 [to the referring clinician] in case I come across it. It really depends on the condition. I can imagine that for onco [genetics], for example, it won’t change [clinical] management, but for many other conditions a class 3 [variant] is often counseled as possibly causative. If you then know for certain that it’s not pathogenic, I think you can report it.” (Participant 7).*

Vos et al. [34] and Solomon et al. [31], among others, have documented the degree to which detected VUS that are communicated to patients as potentially causative of their phenotype can have a significant emotional impact on patients and their families. Furthermore, in some cases, a VUS might be tracked within families to see whether it segregates with the clinical phenotype. Therefore, we suggest that VUS cannot be seen as entities outside of clinical decision-making. In some cases, such as when VUS have previously been communicated to patients as highly suspicious in causing their symptoms, it may be important for the laboratory to report down-classifications of VUS to clinicians. As such, we propose that down-classifications of VUS should not be excluded by default, in contrast to what Chisholm et al. [9] suggest with their proposed workflow for reinterpretation of variants.

### Should reinterpretation, reanalysis or a new test be conducted?

In certain cases, participants believed that reanalysis may prove to be diagnostically more effective than reinterpretation. Reanalysis involves using a patient’s existing raw data (that has been generated as a result of genetic sequencing in the past, e.g. whole exome sequencing) in order to analyze all genes currently associated with the patient’s condition or symptoms, without having to conduct a new genetic test. This includes genes that were not analyzed previously, as a connection with the patient’s condition was not known at the time. Approaches that include novel associated genes have demonstrated the potential to increase diagnostic yield ([30]; Wright et al., [35]). Furthermore, updated workflows and changes in resources (e.g. utilization of a new reference genome [10] or the addition of CNV detection [25]) can sometimes identify variants that previously went unnoticed. In light of fast-changing techniques, improved coverage, decreasing costs, and growing knowledge, it might even be more efficient to request a completely new test (i.e. resequencing of DNA), based on the latest laboratory and analysis standards, rather than performing reinterpretation or reanalysis of existing data.

“*I believe that the patient (in consultation with the clinician) can actively request [reinterpretation], and, in my opinion, in the future, this could [change to] performing a completely new test, instead of reconsidering old data.” (Participant 11).*

Therefore, we recommend that the discussion on reinterpretation in case no genetic diagnosis was made includes the comparison in (cost) efficiency between reinterpretation of previously detected variants, reanalysis of existing data, and redoing genetic tests (i.e. resequencing) based on the latest technologies. To better determine which genes are of interest for reanalysis or a new test, laboratories need to have optimal, recently updated information regarding patient and family phenotypes. For this, continuous feedback is needed from clinicians and patients. Currently, delivery of phenotypic information to the laboratory is considered too brief and unsystematic to reach this goal. Presently, no digital systems are in place to aid in the continuous updating of phenotypic data, before, during, or after the (initial) test. Electronic patient records could help update patient and family phenotypes in a standardized manner, for instance using Human Phenotype Ontology (HPO) or SNOMED CT terminology. Personal health records, which enable patients to digitally manage and update their own medical and family data, could also serve as an important tool in this context [3].

### How are reclassifications perceived and how might this affect laboratory practice?

A final topic raised during the focus group discussion that warrants further investigation in future research is how reclassifications are perceived by laboratory geneticists. For example, are they viewed as the correction of an error that was made in the initial classification or merely as inevitable progression of knowledge? The way reclassifications are perceived may shape the actions taken by laboratory geneticists and their (legal) responsibilities regarding reinterpretation and reclassification of variants. For instance, the degree to which laboratory geneticists need to keep up with evolving evidence on variants within scientific literature.

*“If the classification is downgraded, then immediate contact (…*) *must be sought with the referring clinician, because it possibly concerns a calamity, comparable to entirely missing a mutation. (…*) *I [mean] downgrading of [a class] 5 [variant to a class] 3 [variant]. Of course, [downgrading of a class] 3 [variant to a class] 1 [variant] is not a calamity but rather an insight. [ …*] *When you miss a mutation, you have made an error, and [this is] also [the case] when you classify [a variant] too high.” (Participant 8).*

A United States court case (*Williams* vs *Quest/Athena*^1^), currently in progress, illustrates this issue. Here, a mother (Williams) claims that a genetic misclassification led doctors to administer a pharmaceutical treatment that worsened her son’s condition and caused his death (aged 2, due to an epileptic seizure). A genetic test had been performed in 2007 which had identified a variant in *SCN1A,* a gene implicated in Dravet syndrome. The variant was classified by the laboratory as a VUS and therefore did not affect clinical-decision making by the boy’s clinicians. Two research papers, available publicly at the time of the laboratory report, already stated that this presumed VUS was probably a pathogenic variant. In 2015, the laboratory issued a new report with the variant reclassified as known to be disease causing. The prosecution argues that the initial report contained a mistaken classification and that had this variant been “correctly” classified in 2007, thereby pointing to the diagnosis of Dravet syndrome, different medication would have been given to the child and the fatal seizure would not have occurred [13]. This case raises important questions in the context of variant reclassification. Are reclassifications a correction of a previously made mistake (i.e. the initial classification) in some cases and should laboratory geneticists be held liable for making it, if the knowledge suggesting an alternate classification was available at first interpretation or even afterwards?

A factor complicating the question of error, such as in the *Williams* vs *Quest/Athena* case, is that there is no gold standard regarding what is considered to be enough evidence for an initial classification or for a reclassification. It is not simply a question of whether the evidence for making a certain classification was there and whether it was adequately taken into account. As discussed earlier (Introduction), the amount of evidence needed differs for different conditions and variant types, and what is considered sufficient evidence for a certain variant classification differs between laboratory geneticists. As such, it may be difficult to establish at what point the act of making an initial classification should stray into the territory of error.

Yet, some reclassifications may more likely be considered by laboratory geneticists as the correction of an error in initial classification than others. In the public database ClinVar, variants that used to have a seemingly clear pathogenic or benign classification are currently reclassified or conflicting interpretations are mentioned [20, 28]. This means that in rare cases it is possible that during reinterpretation, pathogenic variants are reclassified to for instance VUS. When a result is initially reported to be pathogenic, reclassification means that confidence regarding its pathogenicity had been unwarranted. Whereas when a result is reported as a VUS, it is implicit that there is currently not sufficient evidence available, but that additional evidence may be obtained in the future, thereby inherently subjecting this variant to possible future reclassification.

We suggest that, rather than considering pathogenic down-classifications or benign up-classifications as constituting an event that was not supposed to happen (i.e. an error), perhaps an increase in awareness is needed that classifications are not necessarily as fixed as they might seem. Currently, this awareness mainly exists for VUS: VUS are expected to change at some point in the future. However, almost all variants are continuously subject to reinterpretation and reclassification. When considering trends on reclassification, it has been shown that progression of information on variants often leads to a shift towards conflicting interpretations of pathogenicity, rather than to more clear and correct classifications [28]. This emphasizes the argument that both initial and reclassifications should be inherently considered as subject to possible change. This does not imply, however, that clinical decisions such as initiating cascade family screening on presumed pathogenic variants should be abolished, based on the argument that one cannot be fully certain that these variants are as pathogenic as we currently think they are. Rather, both healthcare professionals and patients need to be cognizant that evolving knowledge is intrinsic to genetics and that, therefore, changes in a patient’s diagnosis or genetic risk profile are a possibility with increasing knowledge.

## Conclusion

For professionals within clinical genetics laboratories, challenges exist concerning the daily practice of reinterpretation and reclassification of genetic variants, as well as its downstream effects, such as issuing new reports to relevant clinicians. Based on issues noted by representatives from all (nine) Dutch clinical molecular genetics diagnostic laboratories, discrepant practices between laboratory geneticists, and current literature, we have formulated and discussed five challenging questions for daily laboratory practice that need to be addressed in the process of creating and refining guidelines: 1) Should active reinterpretation of variants be conducted by the laboratory as a routine practice?; 2) How does reinterpretation initiated by the laboratory relate to patient expectations and consent?; 3) When should reinterpreted data be considered clinically significant and communicated from laboratory to clinician?; 4) Should reinterpretation, reanalysis or a new test be conducted?; 5) How are reclassifications perceived and how might this affect laboratory practice?

This study shows the importance of looking at laboratory practice and perceptions to identify relevant input for policy and guideline discussions, as well as future research, in the context of managing the changing nature of genetic data. The laboratories in our study reinterpret genetic variants reactively, mainly due to a request by a clinician or upon identification of a previously classified variant in a new patient. This is in line with international guidelines that place no responsibility on laboratories to actively reinterpret genetic data [4, 18]. However, in practice, the topic of how active the laboratory’s role should be in initiating reinterpretation of genetic information and what this would imply for discussions on patient autonomy appeared to be a salient one for laboratory geneticists, with differing opinions on desirability and one laboratory investigating how to implement active, periodic reinterpretation in their practice. Furthermore, our study demonstrates that laboratory geneticists are concerned with the efficacy of patient consent procedures, the emotional effect reclassifications (e.g. VUS down-classifications) might have on patients, and what is defined as clinically significant data; issues that have traditionally been attributed to and investigated within the realm of clinicians and patients. Understanding these concerns as they occur in laboratory practice provides important context for creating and implementing policies, not only around reinterpretation, but also its downstream effects, such as recontacting patients.

This study has a number of important limitations. This study was carried out with participants from the Netherlands and in non-commercial, diagnostic laboratories only, as commercial laboratories are not certified to conduct clinical genetic testing in The Netherlands. It would be interesting to see whether a study with participants from different contexts, such as a commercial laboratory setting or other countries, would yield additional themes and issues.

## Supplementary information

**Additional file 1.** Topic guide used for online focus group discussion with Dutch clinical laboratory geneticists.

## Data Availability

The dataset generated and analysed during the current study is not publicly available as consent was not sought from participants to publicize the full transcript of the online focus group discussion. In case of expressed interest in the dataset, the authors will contact participants in order to ask for their permission to share the dataset. Upon participants’ consent, the dataset will be made available to the requesting party.

## Abbreviations

B: Benign
LB: Likely Benign
LP: Likely Pathogenic
P: Pathogenic
VUS: Variant of Uncertain Significance

## Footnotes

1 The authors do not aim to express any statements or opinions regarding this individual case. Many of its details are inaccessible to the public and more issues are involved in this case than are discussed in this paper. The case is strictly used to highlight the significance and complexity of certain questions regarding reclassification in practice and to stimulate discussion.
