# Supplementary material for: Reinterpretation, reclassification, and its downstream effects: challenges for clinical laboratory geneticists
Source: BMC Med Genomics. 2019 Nov 29;12:170. doi: 10.1186/s12920-019-0612-6 (PMC6883538; doi:10.1186/s12920-019-0612-6)
Supplement: Supplementary file 1 — Additional file 1. Topic guide used for online focus group discussion with Dutch clinical laboratory geneticists. [file 12920_2019_612_MOESM1_ESM.pdf]

Topic list for online focus group discussion*

**Translated from Dutch by LJ and JM*

Day 1 REINTERPRETATION

Day 1: Reinterpretation of VUS: Policy vs practice

Welcome to day 1 of our online discussion!

Today and tomorrow we will discuss REINTERPRETATION of (in particular) VUS. We define reinterpretation as the reassessment of existing genetic results from diagnostic analyses (that have been conducted in the past and in which variants were detected of which the clinical significance is or was unclear). The goal is to determine if these VUS can be reclassified towards (likely) pathogenic or (likely) benign.

This concerns systematic, periodic reinterpretation by the lab, as well as irregular/incidental reinterpretation or reclassification of variants initiated by the lab, clinician, or patient.

This reinterpretation or reclassification may concern individual variants (reclassification based on new knowledge that has been obtained "incidentally" via analysis of new patients, and newly found data from databases and literature in this context) as well as groups of variants.

Even though we will focus on VUS in the following discussion, the discussion may also include other variants that have previously been classified as LP (likely pathogenic) or LB (likely benign), for instance.

The sub questions serve as a guideline for answering the questions/propositions; therefore you don't have to give an extensive answer to every sub question, unless this is your preference, of course.

Question 1: What is your own practical experience with reclassification/reinterpretation of VUS?

Sub questions (guideline for answering the question):

1. Have you ever been involved in reinterpretation/reclassification of VUS?
2. In which cases/for what reasons does/did this happen?
3. Who initiated this?
4. What is/was your method (how did you come to reinterpretation/reclassification)?
5. Which dilemmas or practical bottlenecks or barriers do/did you experience?

Question 2: Does your lab have a formal policy regarding reclassification/reinterpretation of VUS?

Sub questions (guideline for answering the question):

1. If yes, can you describe this policy?
2. If no, why not and how so?
3. How and by whom are priorities determined regarding the policy around reinterpretation/reclassification policy and what is this dependent on?
4. Do clinical geneticists play a role in this process? If yes, what role?
5. Are other medical (organ-)specialists involved in this process (e.g. in MDTs)?

Proposition: in our lab, policies which have (possibly) been documented in protocols regarding reinterpretation/reclassification differ from practice

Sub questions (guideline for answering the question):

1. Do you agree with this proposition?
2. If yes, in what way does it differ, and, in your opinion, why is this?
3. Do you believe it is a problem that policy differs from practice?
4. Can you name specific examples from your practice or your lab's practice (if you don't do practical work yourself)?

Day 2: REINTERPRETATION proposition 1
Day 2: Reinterpretation of VUS: standardization and automation of processes?

Welcome to day 2 of our discussion!

Thank you for the interesting conversation the previous day: this topic will remain open the entire week and you can keep replying.

For the sake of overview, we have decided to split up the questions and propositions.

We define reinterpretation as the reassessment of existing genetic results from diagnostic analyses (that have been conducted in the past and in which variants were detected of which the clinical significance is or was unclear). The goal is to determine if these VUS can be reclassified towards (likely) pathogenic or (likely) benign.

This concerns systematic, periodic reinterpretation by the lab, as well as about irregular/incidental reinterpretation or reclassification of variants initiated by the lab, clinician, or patient.

This reinterpretation or reclassification may concern individual variants (reclassification based on new knowledge that has been obtained "incidentally" via analysis of new patients and newly found data from databases and literature in this context) as well as groups of variants.

Proposition 1: (Ideal) reclassification/reinterpretation processes are strongly dependent on context/symptoms and will always remain as such.

Day 2: REINTERPRETATION proposition 2
Day 2: Reinterpretation of VUS: standardization and automation of processes?

Proposition 2: (Systematic/periodic) reinterpretation processes should be protocolled, (and therefore) standardized and automized, as much as possible.

Day 2: REINTERPRETATION proposition 3
Day 2: Reinterpretation of VUS: standardization and automation of processes?

Proposition 3: The individual laboratory specialist/technician has an important influence on how reclassification/reinterpretation of variants occurs in practice.

Day 3: RECONTACT question 1
Day 3: Recontact based on reinterpretation and/or changes in classification: policy versus practice

Today and tomorrow the discussion will focus on RECONTACT with clinicians, i.e. clinical geneticists and (if applicable) other medical specialists, to communicate new information concerning genome

analyses of which the result was communicated in the past. Emphasis is therefore on the lab's renewed contacting of clinicians and not on recontacting patients (because in the Netherlands this is conducted by clinicians).

Queston 1: What is your practical experience with recontacting clinicians after reinterpretation has led to a change in result?

Sub questions.

1. In which cases/for what reasons does this occur?

2. What dilemmas do you encounter in this context/what barriers do you experience?

Day 3: RECONTACT question 2
Day 3: Recontact based on reinterpretation and/or changes in classification: policy versus practice

Question 2: Does your lab have a formal policy regarding recontacting clinicians after reinterpretation has led to a change in result?

Sub questions:

1. If yes, could you describe this policy as precisely as possible?

Day 3: RECONTACT proposition 1
Day 3: Recontact based on reinterpretation and/or changes in classification: policy versus practice

Proposition 1: in our lab, policies which have (possibly) been documented in protocols regarding recontact differ from practice

Sub questions:

1. Can you name specific examples from your practice or your lab's practice (if you don't do practical work yourself)?

Day 3: RECONTACT proposition 2
Day 3: Recontact based on reinterpretation and/or changes in classification: policy versus practice

Proposition 2: 'clinical actionability' plays an important role in decisions regarding recontact (If reinterpretation has led to a change in classification)

Sub questions:

1. Why does/doesn't it?

2. What do you use as a definition of clinical actionability?

Day 4: RECONTACT question 1
Day 4: Recontact based on reinterpretation and/or changes in classification: dilemmas

Today we will continue our discussion on RECONTACT with clinicians, i.e. clinical geneticists and (if applicable) other medical specialists, to communicate new information concerning genome analyses of which the result was communicated in the past. Emphasis is therefore on the lab's renewed contacting of clinicians and not on recontacting patients (because in the Netherlands [the latter] is conducted by clinicians).

Question 1: When a variant is reclassified in relation to a specific patient, this of importance for unrelated patients/families that have the same variant. Which clinicians (of which patients) does your lab recontact?

Sub questions:

- How/by whom is this decision made?
- What would be ideal practice in this context?

Day 4: RECONTACT proposition
Day 4: Recontact based on reinterpretation and/or changes in classification: dilemmas

Proposition: It is always clear to me and my colleagues/team whether reinterpreted/reclassified genetic information is or is not medically relevant and should be passed on to clinicians.

Sub questions:

1. Why is/isn't that the case?
2. What actions occur when this is not clear?

Day 4: RECONTACT question 2
Day 4: Recontact based on reinterpretation and/or changes in classification: dilemmas

Question 2: Which other (not yet discussed) considerations, dilemmas and problems exist in the context of recontact in laboratory practice?

Day 5: Proposition 1
Day 5: Responsibilities concerning reinterpretation and recontact

Today the topics (REINTERPRETATION and RECONTACT) are combined to map your perspectives on responsibilities and expectations concerning these processes.

Proposition 1: I notice that other parties (patients, clinicians) sometimes have unrealistic expectations of the laboratory in the context of reinterpretation and (therefore) recontact.

Sub questions:

1. If yes, what are these expectations in your practice? Can you name examples?
2. Is there a difference between should, could, and want, in this context?

Day 5: Proposition 2
Day 5: Responsibilities concerning reinterpretation and recontact

Proposition 2: The lab should take more responsibility regarding (initiation of) reinterpretation and recontact in the near future.

Sub questions:

1. Why should/shouldn't it?
2. How should the responsibility for (initiating) reinterpretation and recontact be distributed between laboratory, clinician and patient?

Day 5: Proposition 3
Day 5: Responsibilities concerning reinterpretation and recontact

Proposition 3: When contacting a clinician with new information, I expect that this is always communicated to the patient and other involved medics (specialists/general practitioner).

Sub questions:

1. Why is/isn't this the case?

Day 5: Question
Day 5: Responsibilities concerning reinterpretation and recontact

Question: In your opinion, what more is needed in order for everybody to carry out their role/responsibility well regarding responsible and feasible reinterpretation and recontact processes?

Sub questions:

1. What do you need from/in the interaction with clinicians?

2. Is there a difference between should, could, and want, in this context?
